# Polydextrose changes the gut microbiome and attenuates fasting triglyceride and cholesterol levels in Western diet fed mice

**DOI:** 10.1038/s41598-017-05259-3

**Published:** 2017-07-13

**Authors:** Ghulam Shere Raza, Heli Putaala, Ashley A. Hibberd, Esa Alhoniemi, Kirsti Tiihonen, Kari Antero Mäkelä, Karl-Heinz Herzig

**Affiliations:** 1 Research unit of Biomedicine and Biocenter of Oulu, Department of Physiology, University of Oulu, Oulu, Finland; 2 DuPont Nutrition and Health, Global Health and Nutrition Science, Kantvik, Finland; 3 DuPont Nutrition and Health, Genomics & Microbiome Science, St. Louis, MO USA; 4 Avoltus Oy, Turku, Finland; 5 Department of Gastroenterology and Metabolism, Poznan University of Medical Sciences, Poznan, Poland; 6 Medical Research Center (MRC), University of Oulu, and University Hospital, Oulu, Finland

## Abstract

Obesity and dyslipidemia are hallmarks of metabolic and cardiovascular diseases. Polydextrose (PDX), a soluble fiber has lipid lowering effects. We hypothesize that PDX reduces triglycerides and cholesterol by influencing gut microbiota, which in turn modulate intestinal gene expression. C57BL/6 male mice were fed a Western diet (WD) ±75 mg PDX twice daily by oral gavage for 14 days. Body weight and food intake were monitored daily. Fasting plasma lipids, caecal microbiota and gene expression in intestine and liver were measured after 14 days of feeding. PDX supplementation to WD significantly reduced food intake (p < 0.001), fasting plasma triglyceride (p < 0.001) and total cholesterol (p < 0.05). Microbiome analysis revealed that the relative abundance of *Allobaculum*, *Bifidobacterium and Coriobacteriaceae* taxa associated with lean phenotype, increased in WD + PDX mice. Gene expression analysis with linear mixed-effects model showed consistent downregulation of *Dgat1*, *Cd36*, *Fiaf* and upregulation of *Fxr* in duodenum, jejunum, ileum and colon in WD + PDX mice. Spearman correlations indicated that genera enriched in WD + PDX mice inversely correlated with fasting lipids and downregulated genes *Dgat1*, *Cd36* and *Fiaf* while positively with upregulated gene *Fxr*. These results suggest that PDX in mice fed WD promoted systemic changes via regulation of the gut microbiota and gene expression in intestinal tract.

## Introduction

Western diets high in saturated fat and processed meat are strongly implicated in the increasing prevalence of obesity, diabetes and cardiovascular diseases (CVD)^1^. Dyslipidemia is characterized by increased levels of plasma triglyceride-rich lipoproteins and non-HDL-cholesterol and reduced concentration of HDL cholesterol^2^. It affects almost 50% of individuals in Western populations, and is a hallmark for metabolic syndrome and CVD^3, 4^. Lifestyle management and healthy dieting habits, including reduced consumption of saturated fats as well as increased dietary fiber and unsaturated fats, are recommended as the first therapeutic choice for CVD and dyslipidemia^2, 5^. The influence of dietary fibers on dyslipidemia has been demonstrated in both clinical and animal settings, demonstrating the hypocholesterolemic properties of soluble fibers^6, 7^.

The gut microbiota has been recognized as one key factor influencing whole-body metabolism by affecting energy balance and driving metabolic diseases by its suggested stimulation of low-grade inflammation^8^. In the Western countries, the intake of dietary fibers is lower than the recommended 25–30 g/d^9^. The low level of dietary fiber can significantly alter the gut microbiome, which has been suggested to result in a dysregulation of metabolism^8^. The five prevalent phyla of gut microbiota in mammals include Firmicutes, Bacteroidetes, Actinobacteria, Proteobacteria and Fusobacteria^10^. Dysbiosis of gut microbiota has been suggested to associate with the occurrence of chronic diseases such as inflammatory bowel disease (IBD), obesity and type 2 diabetes^11^. High-fat, high-sugar Western diet has been shown to promote a shift in gut microbiota composition toward high numbers of Clostridia and significant decrease in Bacteroidetes^12^. Moreover, high-fat diet has been observed to promote a reduction of Bacteroidetes and an increase of Firmicutes and Proteobacteria, in both obese and lean phenotypes^13^. Selective modulation of the gut microbiota and metabolic pathways by fermentation of prebiotic fiber and production of short chain fatty acids (SCFA) can affect systemic fatty acid metabolism through modulating host gene expression and providing a substrate for cellular catabolic and anabolic reactions^14^.

Polydextrose (PDX) is a non-viscous soluble fiber, partially fermented by the gut microbiota. PDX can partially or totally replace sugar, fat or starch and can be presented as reduced fat, reduced sugar, low calorie, or even low glycemic index, depending upon the applications^15^. As a fermentable soluble fiber, PDX promotes the growth of bifidobacteria and lactobacilli while preventing the growth of detrimental bacteria, e.g. Clostridia, in addition to increasing SCFA production^16–18^.

PDX has been demonstrated to reduce both total and LDL cholesterol in hypercholesterolemic healthy patients and in patients with impaired glucose metabolism^19, 20^. However, the exact mechanism of how PDX exerts these hypolipidemic effects has remained unclear.

Therefore, we studied in a mouse model if PDX reduces blood triglyceride and cholesterol by modulating the gut microbiota and by regulating expression of key genes related to the lipid metabolism.

## Results

### Body weight, food intake and plasma lipids

**Figure 1:**
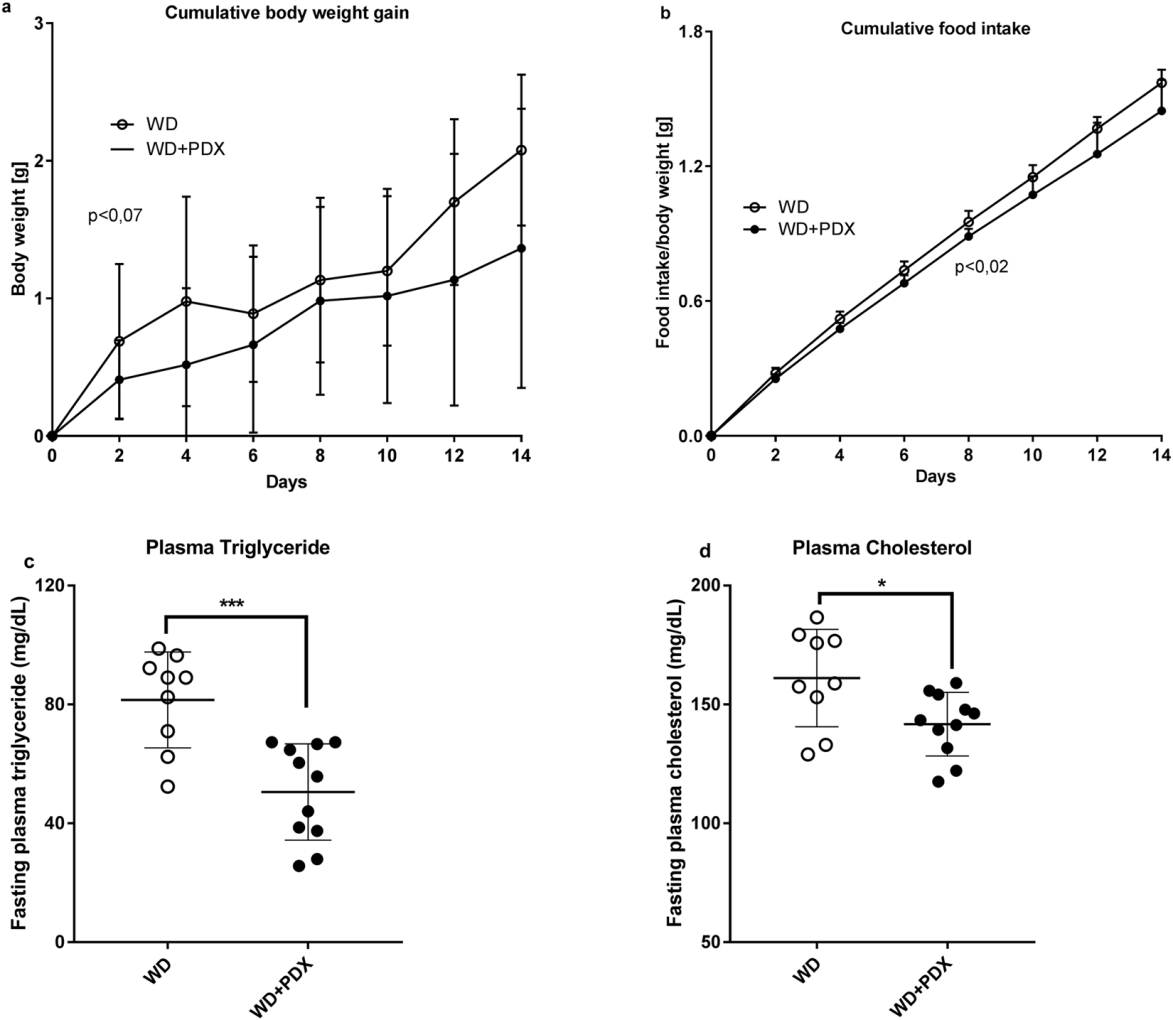
Body weight gain (**a**), Cumulative food intake (**b**), fasting plasma triglyceride and cholesterol (**c** and **d**). WD; Western diet fed mice, WD + PDX; Western diet fed mice that received PDX 75 mg twice daily for 14 days. The values are presented as mean ± SD. p < 0.05 was considered as statistical significance. *p ≤ 0.05, **p ≤ 0.01, ***p ≤ 0.001.

Over the two-week test period the mice did not significantly change their body weight (Fig. 1a) (p = 0.07), even though a significant (p < 0.001) reduction in the cumulative food intake was observed in the WD + PDX mice (Fig. 1b).

Fasting plasma triglyceride (50 ± 4.8 mg/dL; 81 ± 5.3 mg/dL p < 0.001) and plasma cholesterol (141 ± 4 mg/dL; 161 ± 7 mg/dL, p < 0.05) were significantly reduced in WD + PDX mice compared to WD, respectively (Fig. 1(c and d)). In addition epididymal fat weight was significantly reduced (p < 0.05) and there was also a significant increase (p < 0.0001) in caecal content weight of the WD + PDX animals compared to the WD (Supplementary Fig. S1a-c).

### Faecal fat content

The faecal fat content was measured from pooled faeces samples from WD and WD + PDX. The amount of fat in the feces was comparable between the two samples at the start of the experiment (Day 0): WD 4.4 g fat/100 g feces while WD + PDX mice had fat 4 g/100 g feces (Supplementary Fig. S2). On day 14 the amount of fat in the feces from the WD was 5.9/100 g while in WD + PDX mice the amount of fat was increased numerically to 6.8 g fat/100 g feces. Statistical analysis was not performed as only pooled samples were measured.

### Gene expression in intestine and liver

**Figure 2:**
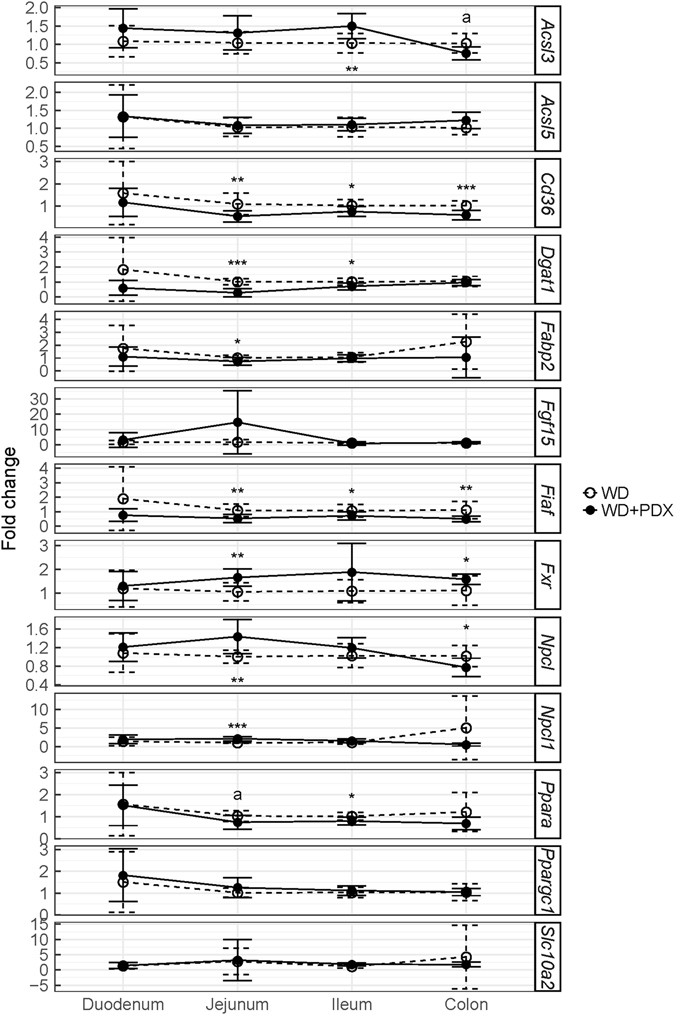
Gene expression analysis of *Acsl3*, *Acsl5*, *Cd36*, *Dgat1*, *Fabp2*, *Fgf15*, *Fiaf*, *Fxr*, *Npcl*, *Npcl1*, *Ppara*, *Ppargc1* and *Slc10a2* in duodenum, jejunum, ileum and colon. *Dgat1*, *Cd36*, *Fiaf* and *Fxr* showed consistent statistically significant differences between WD and WD + PDX mice in jejunum, ileum and colon. *Npcl1*, *Npc1*, *Acsl3*, *Fabp2* and *Ppara* showed statistically significant difference either in jejunum or ileum or colon between WD and WD + DX mice. The letter “a” represents nearly significant changes. The values are presented as mean ± SD. *p ≤ 0.05, **p ≤ 0.01, ***p ≤ 0.001.

Four genes (*Dgat1*, *Cd36*, *Fiaf* and *Fxr*) had constant expression differences in more than one part of the intestinal tract (jejunum, ileum and colon) when PDX + WD was compared to WD (Fig. 2). *Cd36* and *Fiaf* expression were significantly reduced in jejunum, ileum and colon while *Dgat1* was reduced only in jejunum and ileum. *Fxr* was significantly upregulated in jejunum and colon. *Npc11*, Npc1, *Acsl3*, *Fabp2* and *Ppara* were also differentially regulated expression in the different parts of the small intestine: *Npcl1* and *Npc1* upregulated in jejunum, *Acsl3* upregulated in ileum and *Fabp2* downregulated in jejunum, Furthermore, PPARα expression was nearly significantly reduced by PDX in jejunum (p = 0.0523) and significantly in ileum (p = 0.0434).

In the liver fatty acid metabolizing genes (*Hmgcr*, *Ldlr*, *Lpl*, *Acot3*, *Prkaca*, *Prkaa*, *Acat1*, *Acot2*, *Cyp7a1*, *Slc27a2*, *Acot6*, and *Cd36*) were not statistically different between the groups (Supplementary Fig. S3). There was a trend for upregulation in genes *Hmgcr* (p = 0.07), *Ldlr* (p = 0.06) and *Acot3* (p = 0.09) in WD + PDX compared to WD.

### Characterization of caecal microbiota

#### Alpha diversity

**Figure 3:**
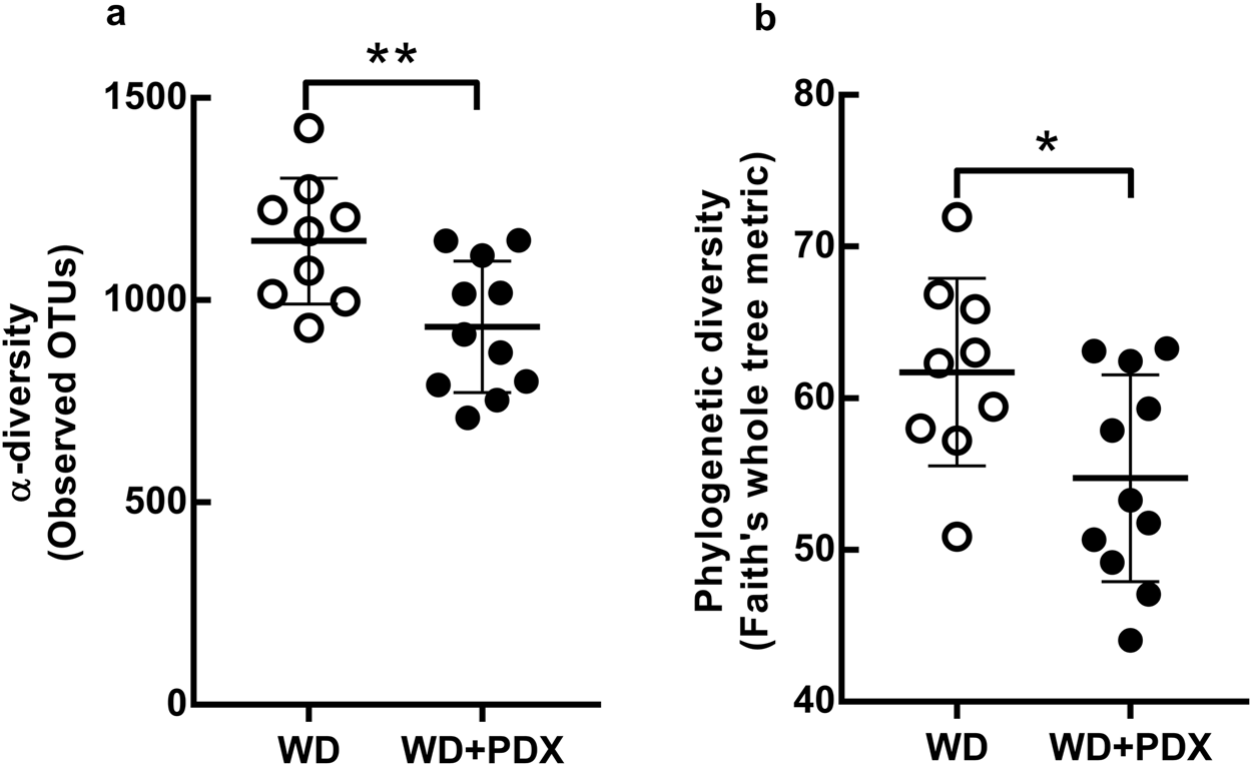
Alpha diversity metrics; (**a**) observed OTUs and (**b**) phylogenetic diversity for caecal microbiota in WD and WD + PDX mice. The values are presented as mean ± SD. *p ≤ 0.05, **p ≤ 0.01; non-parametric t-test using 1000 Monte Carlo permutations.

Alpha diversity (species richness) was measured by the number of observed OTUs per sample and by the Phylogenetic Diversity Whole Tree metric which weights OTUs by their phylogenetic distance. The alpha diversity rarefaction curves suggested the sequencing depth was sufficient to capture the biodiversity in the samples (Supplementary Fig. S4). Mice fed WD + PDX had a lower alpha diversity compared to WD, based on the Observed OTUs (p = 0.003) (Fig. 3a) and Phylogenetic Diversity Whole Tree (p = 0.036) (Fig. 3b) metrics.

#### Microbiota communities

**Figure 4:**
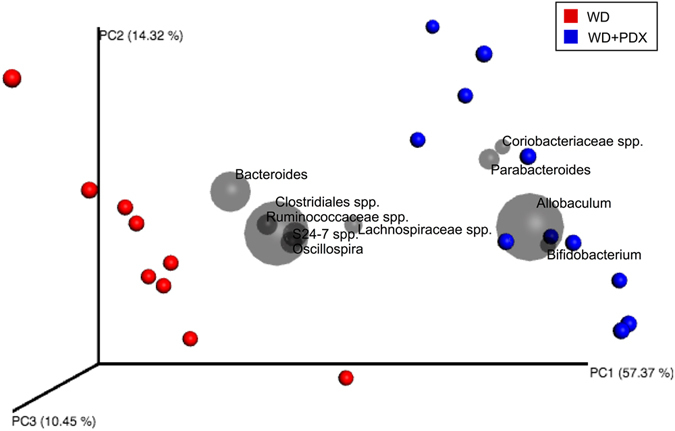
Principal coordinates analysis (PCoA) for weighted UniFrac distance metric in caecal microbial communities from WD and WD + PDX mice. The top 10 most abundant taxa are plotted using grey circles. The circle size is proportional to abundance of each genus, thereby illustrating taxa that are driving differentiation between the microbial communities. Sample clustering by diet is significant p < 0.001 (PERMANOVA).

PCoA plots with weighted UniFrac matrices (Fig. 4) and unweighted UniFrac matrices (Supplementary Fig. S5) show a clear separation of microbial communities in mice fed with the different diets. The community composition was relatively consistent within the groups, and sample clustering by treatment was significant (PERMANOVA, p = 0.001). The ten most prevalent genera were plotted to identify those taxa that were most responsible for the observed sample clustering by treatment. *Bacteroides*, *Clostridiales* spp., *Ruminococcaceae* spp., *Lachnospiraceae* spp., *Oscillospira* and *S24*-*7* spp. were enriched in WD mice, while *Coriobacteriaceae* spp., *Parabacteroides*, *Allobaculum* and *Bifidobacterium* were abundant in the WD + PDX mice.

#### Relative abundance of caecal microbiota

Caecal microbiota in all samples at phylum level was dominated by Firmicutes (59.7% abundance) followed by Bacteroidetes (25.9% abundance), Actinobacteria (6.5%) and Proteobacteria (4.3%), whereas minor phyla were Deferribacteres (1.7%) and Verrucomicrobia (1.6%).

**Figure 5:**
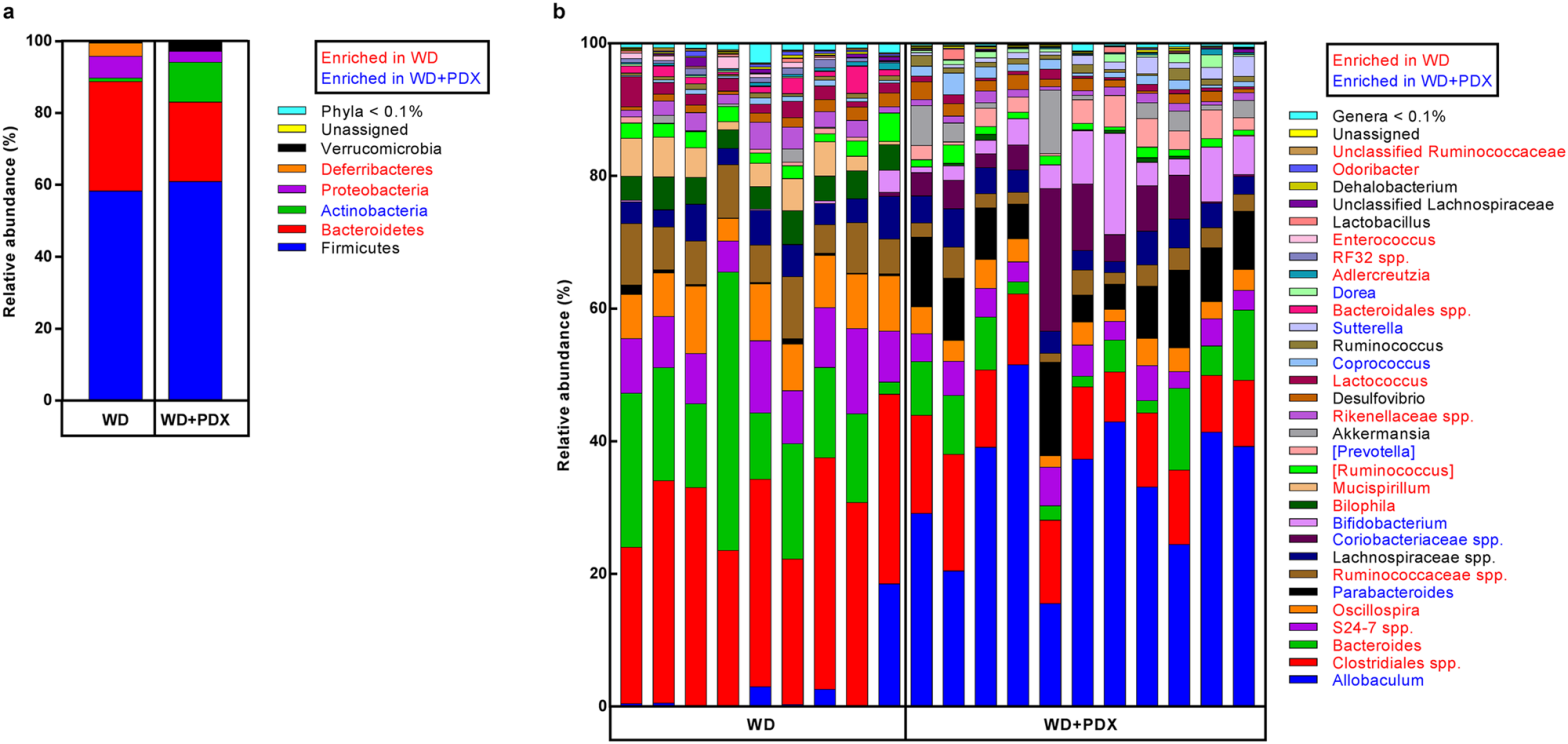
Relative abundance of caecal bacteria at (**a**) phylum and (**b**) genus level of taxonomy in WD and WD + PDX mice. Square brackets indicate the Greengenes database notation for proposed taxonomy. Blue colored taxa were significantly enriched in WD mice and red colored taxa are significantly enriched in WD + PDX mice. p < 0.05; Mann-Whitney U test with false discovery rate correction.

At phylum level, great differences between the treatments were observed (Fig. 5a). The most substantial effect was an increase of Actinobacteria in WD + PDX mice (11.0% abundance) compared to WD-fed mice (1.0% abundance, p < 0.005). The relative abundance of Firmicutes was comparable between the two groups, but at the same time, a significant decrease in the relative abundance of Bacteroidetes was observed in WD + PDX mice (22.1% in WD + PDX versus 30.5% in WD mice, p = 0.023), which led to a significant increase in the Firmicutes:Bacteroidetes ratio in WD + PDX mice (p = 0.0379). Moreover, the minor Deferribacteres, were practically absent in WD + PDX mice compared to WD (0.02% in WD + PDX versus 3.7% in WD mice p < 0.005). Additionally, a significant decrease in the phylum Proteobacteria was observed in WD + PDX mice (3.0% abundance) compared to WD mice (6.0% abundance, p < 0.005). Polydextrose treatment in Western diet also increased the relative abundance of Verrucomicrobia (2.6% in WD + PDX versus 0.42% in WD), but this was not quite significant (p = 0.0611).

The most prominent changes at genus level were observed within the Firmicutes, despite no phylum level difference between the groups (Fig. 5b). Within this phylum, *Allobaculum* was highly enriched in the WD + PDX mice (34%) compared to WD mice (2.85%) (p < 0.001), while *Clostridiales* spp., was decreased in WD + PDX mice (11.5% versus 28.9%, p < 0.001). *Oscillospira* (3.2% versus 7.4%, p < 0.005) and *Ruminococcus* spp. (2.7% versus 6.9%, p < 0.001) were also decreased in WD + PDX mice. Within Actinobacteria, the relative abundance of unknown genus of *Coriobacteriaceae* spp. and *Bifidobacterium* were increased in WD + PDX mice (5.7% and 5.1%, respectively) compared to WD (0.08%, 0.46%, respectively, p < 0.01 in both). Relative abundance of the genus *Parabacteroides* from phylum Bacteroidetes was increased in WD + PDX (8.3%) compared to WD (0.4% p < 0.001). *Bilophila* (phylum Proteobacteria) was decreased in WD + PDX mice (0.27% versus 3.9%, p < 0.001), and low abundance genera *Sutterella* was increased over 92-fold in WD + PDX mice compared to WD mice (1.2% vs 0.01% p < 0.001). From phylum Deferribacteres that were almost completely absent from WD + PDX mice, the genus *Mucispirillum* (0.027% versus 3.7%, respectively, p < 0.001) was enriched over 136-fold in WD mice. Additionally, there was over 6-fold increase in the relative abundance of genus *Akkermansia* (phylum Verrucomicrobia) in the WD + PDX mice (2.6%) compared to WD (0.42%), but not significant (p = 0.0559). Minor changes were observed in several other genera, as shown in (Fig. 5b).

#### Correlation of caecal microbes with plasma lipids

**Figure 6:**
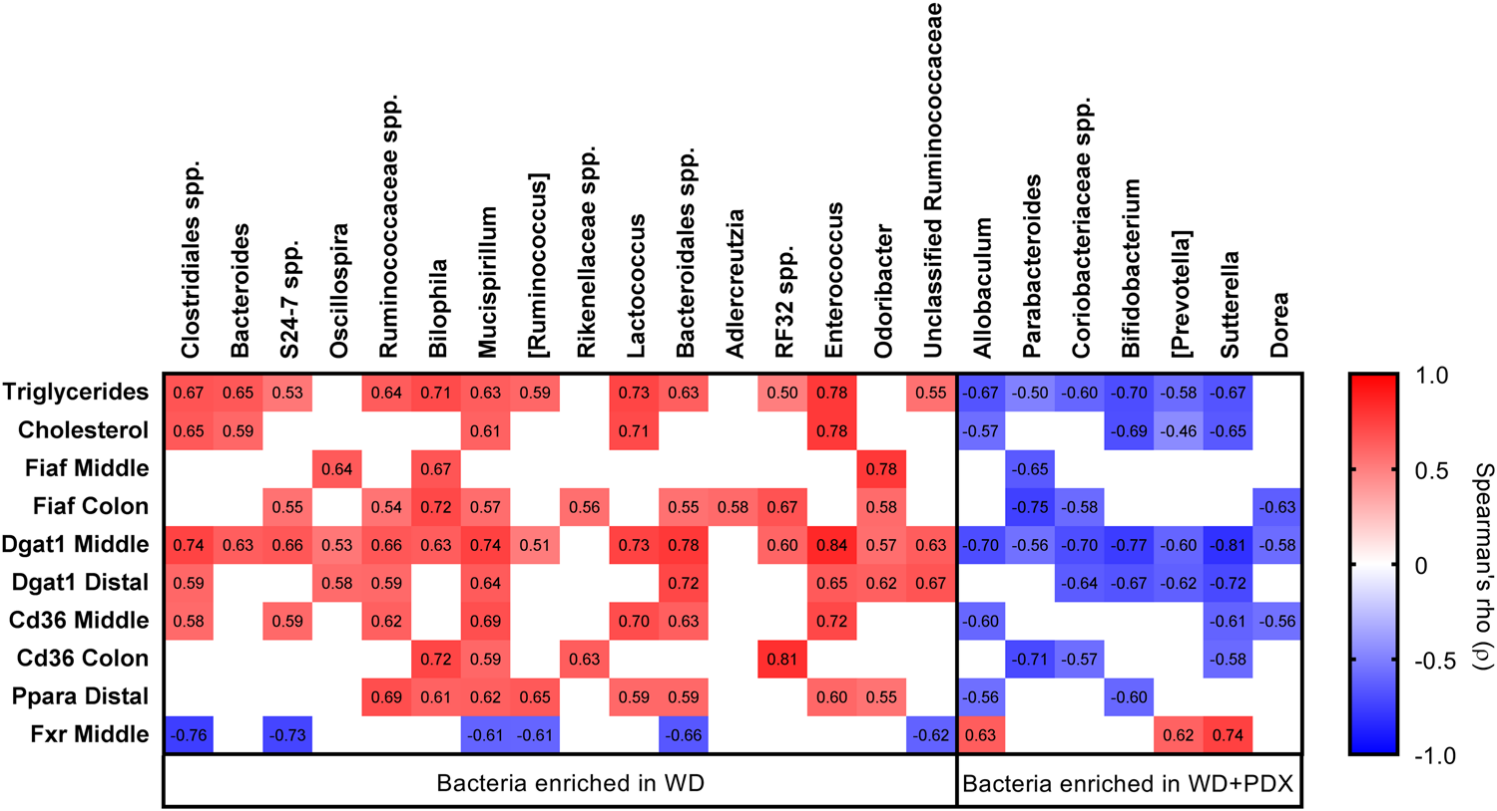
Spearman correlation coefficients of caecal microbial genera with lipid parameters and gene expression. Square brackets indicate the Greengenes database notation for proposed taxonomy. Only correlations that were significant after false discovery rate correction are shown, p < 0.05.

A spearman correlation analysis was performed to assess association of caecal microbial genera and lipid parameters (Fig. 6). Both plasma triglycerides and total cholesterol values correlated inversely with *Bifidobacterium*, *Allobaculum*, *Sutterella* and [*Prevotella*] (Greengenes database notation for proposed taxonomy). Plasma triglyceride values also correlated inversely with *Coriobacteriaceae* spp. and *Parabacteroides*. The genera that correlated negatively with the plasma lipids were all enriched in WD + PDX mice. Positive correlation, for both triglyceride and total cholesterol was observed to genera that were enriched in WD mice: *Enterococcus*, *Lactococcus*, *Mucispirillum*, *Bacteroides* and *Clostridiales* spp., and in addition, triglyceride values correlated positively to *Bilophila*, *Ruminococcaceae* spp., [*Ruminococcus*], unclassified *Ruminococcaceae*, *Bacteroidales* spp., *S24*-*7* spp. and *RF32* spp., all enriched in WD mice.

#### Correlation of plasma lipid and caecal microbes with gene expression

*Acsl5* and *Cd36* in jejunum positively correlated with the triglyceride (Spearman ρ = 0.8536, p < 0.01 and ρ = 0.7029, p < 0.05, respectively), while cholesterol only with *Acsl3* in colon (ρ = 0.7, p < 0.05) of WD mouse. Jejunal *Npcl1* and *Acsl3* expression was inversely correlated to plasma triglyceride (ρ = −0.6241, p < 0.05 and ρ = −0.6515, p < 0.05, respectively) and plasma cholesterol (ρ = −0.7545, p < 0.01 and ρ = −0.6818, p < 0.05, respectively) in WD + PDX mouse. Gene expression in intestine was correlated with caecal microbes (Fig. 6). *Fiaf*, *Dgat1* and *Cd36*, in jejunum, or in ileum or in colon were correlated positively to genera that were enriched in WD mice and negatively to genera that were enriched in WD + PDX mice. Furthermore, *Ppara* in ileum showed a similar trend. The only exception to this rule was jejunal *Fxr* expression, which showed the opposite trend. Even though we did not find any significantly differentially regulated genes in liver tissue, *Prkaa*, *Hmgcr*, *Acat*, *Ldlr*, *Lpl*, *Slc27a2*, *Acot3*, *Ppara*, and *Ppargc1* showed inverse correlation with plasma triglyceride in WD + PDX while in WD there was no correlation (Supplementary Table S2). *Lpl* was the only gene that showed similarly an inverse correlation to the plasma total cholesterol in WD + PDX mice while no correlation was observed in WD mice (Supplementary Table S2).

## Discussion

PDX supplementation to Western diet fed mice significantly reduced food intake, fasting plasma triglyceride, and total cholesterol and epididymal fat mass. Furthermore, microbiome analysis revealed a clear effect of PDX with increase in relative abundance of genera *Allobaculum*, *Bifidobacterium*, and *Coriobacteriaceae* spp. suggested to be associated with a lean phenotype and host lipid metabolism^21–23^. Gene expression analysis with a linear mixed-effects model showed a consistent downregulation of *Dgat1*, *Cd36* and *Fiaf* and upregulation of *Fxr* in several intestinal segments either in duodenum, jejunum, ileum or colon in WD + PDX mice. Furthermore, in liver the significant inverse Spearman correlation between several genes related to lipid metabolism *Prkaa*, *Hmgcr*, *Acat*, *Ldlr*, *Lpl*, *Slc27a2*, *Acot3*, *Ppara*, and *Ppargc1* and plasma triglyceride and between *Lpl* and plasma total cholesterol, indicates that polydextrose ingestion has metabolic effects in liver regulating circulating lipoproteins.

The decrease in food intake with PDX in Western diet fed mice might be due to its satiety effect; satiety effect and reduction of energy intake of PDX has already been reported in humans^24, 25^. PDX is a complex polymer and its fermentation and production of SCFA continues to the distal part of the colon, as residues of undigested PDX have been found from feces of subjects consuming PDX^26^. High-fat diet has been shown to suppress the formation of SCFAs and dietary fiber counteracting this change^27^. Fermentable carbohydrates and SCFAs have been demonstrated to protect against diet-induced obesity and inhibit food intake via increase in the circulating concentrations of the anorectic gut hormones, glucagon-like peptide-1 (GLP-1) and peptide YY (PYY)^28, 29^ which has also been observed with polydextrose in human studies with respect to GLP-1^30, 31^. Interestingly, the genes consistently regulated along the intestinal tract in mice by the inclusion of PDX in the Western diet are known to be regulated by the SCFAs or by the gut microbiota. Butyrate and propionate have been shown to stimulate FIAF production and its cleavage in several studies^32, 33^ and there are indications that butyrate could regulate CD36 expression^34^. Soluble metabolites of specific bacteria strains can enhance *Fxr* gene expression and alleviate weight gain in diet-induced obesity^35^. In addition, *Dgat1* expression can be regulated by dietary fiber as well as by SCFA^36, 37^. SCFA administration has been observed to protect from obesity in mice during high-fat feeding and prevented weight gain in overweight adult humans and mice^38–40^.

Previous studies have indicated that PDX-derived microbial metabolites regulate gene expression^41, 42^. Among the genes regulated by PDX are key players in the metabolism of lipids. It has been suggested that gut microbiota alter fat storage through the regulation of *Fiaf* (fasting-induced adipose factor, also known as angiopoietin-like 4 protein, ANGPTL4), an inhibitor of lipoprotein lipase (LPL)^43^. FIAF, produced by brown and white fat, liver and intestine, inhibits LPL that catalyzes uptake of circulating lipids into tissues like skeletal muscle, heart and adipose tissue, regulating fatty acid oxidation in both muscle and adipose tissue^43^. However, it seems that intestinal production of *Fiaf* does not have a role in the gut microbiota-mediated effects on fat storage as intestinal expression of *Fiaf* was elevated in both germ-free and conventional C3H mice on either high-fat or Western diets, without affecting circulating levels of the protein^43, 44^. Therefore, reduction of intestinal *Fiaf* expression by polydextrose indicates that it is not mediating the observed effects in systemic lipids. In turn, *Cd36* expression has been observed to correlate positively with fatty acid absorption and it has been shown that fatty acids uptake is reduced in the *Cd36* null mouse^45^. Recently, it has been suggested that *Cd36* might act as a lipid sensor optimizing the formation of large chylomicrons in the small intestine^46^. Additionally, we found a significant downregulation of *Dgat1* expression in the intestine with PDX supplementation. *Dgat1*-deficient (*Dgat1* ^*−/−*^) mice are lean, resistant to diet-induced obesity and show decreased DGAT activity^47^. Furthermore, it has been shown that obesity resistance of *Dgat1* ^*−/−*^ mice was due to the absence of intestinal *Dgat1* expression^48^. *Dgat1* ^*−/−*^ mice have decreased *PPar*alpha, gamma and delta expression^49^. We also observed reduced *Ppara* expression in the intestine with PDX supplementation.

Another important regulator of triglyceride and cholesterol homeostasis is FXR, which we found significantly upregulated in the ileum and colon by polydextrose. Elevated plasma triglyceride and cholesterol levels have been shown from *Fxr* ^−/−^ knockout mice while a chemical FXR agonist (INT-747) reduces plasma triglyceride and cholesterol^50, 51^. *Zhang*, *et al*.^35^ showed that soluble metabolites of specific strains of bacteria enhance *Fxr* gene expression, alleviating weight gain in diet-induced obesity, and decreasing biochemical markers of liver injury and lipid metabolism^35^.

We saw a trend in upregulation of *Hmgcr*, *Ldlr*, and *Acot3 ﻿in liver samples of PDX supplemented mice*, and significant inverse correlations between several lipid metabolism related genes and plasma triglyceride and total cholesterol ﻿values. SCFA are generally metabolized in colon and liver, affecting gut and liver function^14^. Feeding of SCFA along with high-fat diet in mice for 12 weeks increased the energy expenditure and promoted fatty acid oxidation with expense of carbohydrate oxidation, reducing weight gain in these mice^40^. Based on our results, it is very plausible that polydextrose supplementation in the diet and its fermentation in colon could affect the lipid metabolism in liver through gene expression regulation, even though we observed only trends as well as significant inverse correlations between the gene expression and plasma lipid values. It might be that a more long-term study is needed to see the full-scale changes in liver lipid metabolism upon PDX ingestion. In addition, studies investigating the changes at protein level are needed to verify whether the changes in gene expression correlates to changes in protein levels.

We found that WD + PDX resulted in a significant change of the microbiome with a less diverse microbiota compared to WD mice. In humans, inclusion of PDX as part of a snack bar did not decrease the microbial diversity^26^. Reduced microbial diversity have been reported in some studies on obesity and metabolic syndrome but differences in host species and the complexity of interactions of the microbiota with diet, age genetics, and host environment have been reported as confounding factors^52–54^. We observed that the most affected phyla were Deferribacteres, Actinobacteria, Proteobacteria and Bacteroidetes, and from these Actinobacteria were enriched in the WD + PDX mice while the others were enriched in WD mice. In rodents, an increased ratio of Firmicutes to Bacteroidetes has been proposed as a characteristic of gut bacteria during obesity, however, subsequent studies have shown inconsistent results which was also observed in our study^55, 56^. Factors such as age, site of sampling, genetics, bacteriophages, and environment can affect the gut microbiota composition, explaining some of the discrepancies among the studies^10, 57^.

The most prominently enriched genus in PDX-fed mice was *Allobaculum*, which is often depleted in obese mice and correlates positively with plasma HDL cholesterol^21, 58^. Interestingly, increase in *Allobaculum* has been observed in another study at which the soluble fermentable fiber was fed in a HFD mouse model, providing further evidence that a fermentable fiber can increase the relative abundance of this genus^59^. Furthermore, the enrichment of *Bifidobacterium* in PDX-fed mice is interesting, as low abundance of *Bifidobacteriaceae* has been noted during high fat diet feeding, and associated with development of obesity^22, 60^. Prebiotic supplementation in mice can selectively increase bifidobacteria and concomitantly decrease inflammatory markers and endotoxemia, which are associated with an enhancement in gut barrier function^22^. Prebiotic stimulation of bifidobacteria correlated with an improvement in glucose metabolism as well as decrease in body fat mass^22^. We observed that epididymal fat mass was reduced significantly in PDX fed mice. In humans, PDX has been shown to reduce body fat mass when it is administered together with *Bifidobacterium animalis* ssp. *lactis* 420, even though it did not reduce it on its own^61^. Our findings supports the combination effect of PDX and bifidobacteria in the regulation of body fat mass. *Coriobacteriaceae* has been found to be depleted in type 2 diabetes, suggesting a role in metabolic disorders^23, 62^. In contrast, the abundance of taxa associated with high-fat feeding, such as the family of *Clostridiales* spp. and members of the families *Ruminococcaceae*, *Rikenellaceae*, *Desulfovibrionaceae* and *Deferribacteraceae* ^12, 63–67^ were decreased with PDX supplementation. *Bilophia* (family *Desulfovibrionaceae*) produces hydrogen sulfide, which can cause gut barrier dysfunction, and increase intestinal permeability and inflammation^64^. The high fat feeding has been shown to increase the abundance of phylum Deferribacteres, especially the abundance of the genus *Mucispirillum*, which was enriched in Western diet fed mice in our study^21^. Thus, inclusion of polydextrose seems to exert effects in several members of the gut microbiota, which have been linked to host metabolism.

In conclusion, we found a positive hypolipidemic effect of PDX supplementation in mice during Western diet feeding. The results obtained from the gut microbiota sequencing strengthen the concept that certain bacteria are linked with a Western diet and that the inclusion of PDX caused an enrichment of *Allobaculum*, *Bifidobacterium*, and *Coriobacteriaceae*, and a reduction in bacteria previously linked to high fat feeding. The alteration in the gut microbiota with PDX supplementation was associated with a differential expression of genes such as *Fiaf*, *Dgat1*, *Cd36* and *Fxr*, which are known to be regulated by SCFAs, produced during the fermentation of PDX. There are strong indication that polydextrose could modify hepatic gene expression. We suggest that the systemic hypolipidemic effect of PDX with reduction in epididymal fat is exerted via diet-directed modification of gut microbiota during Western-diet feeding that is associated with favorable changes in the metabolic gene expression.

## Materials and Methods

Animal experiments were conducted in accordance with the guidelines set by the European Community Council Directives 86/609/EEC and were approved by Institutional Animal Care and Use Committee of the Provincial Government. All methods are in compliance with the national guidelines.

Inbred 10-weeks old C57BL/6NCRl male mice (NWD = 9 and NWD + PDX = 11) were purchased from the laboratory animal center of Oulu University, Finland. Mice were housed in individual cages with ad libitum food and water and kept in 21 ± 2 °C and relative humidity 40–60% with a 12 h light and dark period, with lights on at 6 pm. After 1-week of acclimatization, mice were fed Western diet formula D12079B (Research diet Inc NJ, USA) and orally dosed with PDX (Litesse﻿® Ultra, DuPont) 75 mg in water (referred as WD + PDX group) or water alone as control (referred as WD group) twice daily at 8 am and 6 pm for 14 days. The Western diet contains protein 17% Kcal, carbohydrates 43% Kcal, fat 41% Kcal and 5% fiber and 0.21% cholesterol. Body weight and food intake of all the animals were recorded on daily basis. No diarrhea was observed in mice with PDX during feeding trial. Duration of feeding was chosen based on previously published results^68^.

### Tissue and blood sampling

After a twelve-hour fast terminal blood samples were collected from mouse under isofluorane anesthesia in Ethylenediaminetetraacetic acid (EDTA) tubes and sacrificed immediately thereafter by cervical dislocation. EDTA blood was centrifuged for 8000 rpm for 7 min at 4 °C, and the plasma was stored at −70 °C until further measurements. Total cholesterol and triglycerides were measured using DiaSys reagents according to the manufacturer’s instructions (DiaSys Diagnostic System GmbH, Holzheim Germany). Liver, epididymal fat pad, small intestine (duodenum, jejunum and ileum) and colon as well as the contents of the cecum were collected. The wet weight of liver and epididymal fat pad were measured immediately after tissue isolation, and the tissues were immersed in RNAlater solution (Thermo Fischer Scientific, Waltham, MA, US) and stored at −70 °C until further analysis. Caecal contents were transferred in 1.5 ml pre-weighed Eppendorf tubes, wet weight recorded, snap frozen in liquid N2 and stored at −70 °C until used for 16S rRNA sequencing.

### Faeces collection and faecal fat content

Faeces from the mice in the beginning at day 0 and after 14 days of feeding were collected. Mice were placed in new cages and after 24 hours faeces were gathered, pooled and stored at −70 °C. Faecal fat content was analysed using an accredited gravimetric method by acid hydrolysis with 3 M hydrochloric acid by boiling 45 min and solvent extraction (Eurofins Scientific Finland Raisio (Food & Agro), Raisio, Finland).

### Gene expression analysis in liver and intestine

The gene expression analysis was performed using real-time PCR. Briefly, total RNA was isolated from the small intestine (duodenum, jejunum and ileum), colon and liver with Total RNA NucleoSpin 96 RNA kit (Macherey Nagel GmbH & Co. KG, Düren, Germany). RNA concentrations were determined with Qubit 3.0 Fluorometer (ThermoFisher Scientific) and cDNAs synthesized according to the manufacturer’s instructions using SuperScript III and random primers (Thermo Fisher Scientific). The concomitant relative gene transcript analyses were done from triplicates (7500 FAST Real-Time PCR System, Thermo Fisher Scientific) using specific TaqMan Gene Expression Assays. Fatty acid metabolism gene array was performed in liver tissues using PAMM-007Z kit from Qiagen and most promising candidates from array were selected for RT-PCR analysis^41^. The genes for intestinal tissues *Acsl3*, *Acsl5*, *Cd36*, *Dgat1*, *Fabp2*, *Fgf15*, *Fiaf*, *Npcl*, *Npcl1*, *Nr1h4*, *Ppara*, *Ppargc1* and liver tissues *Prkaca*, *Prkaa*, *Hmgcr*, *Acat*, *Acot2*, *Cyp7a1*, *Ldlr*, *Lpl*, *Slc27a2*, *Acot3*, *Acot6*, *Cd36*, *Ppara* and *Ppargc1* gene expression analyses (Thermo Fisher Scientific) (Supplementary Table S1). *Rplp0* was used as a reference gene for both intestinal and liver tissues, which showed consistent expression over the different samples (data not shown).

### Barcoded 16S rRNA amplicon sequencing

Microbial DNA was extracted from the caecal digesta by a bead-beating step before using the QIAamp DNA stool Mini extraction kit (Qiagen, Hilden, Germany). The microbial community composition was analyzed using high throughput amplicon sequencing as previously described^69^. Briefly, the V4 region of the 16S rRNA gene of Bacteria and Archaea was amplified in triplicate PCR with primers 515 F (GTGCCAGCMGCCGCGGTAA) and 806R (GGACTACHVGGGTWTCTAAT) with the addition of appropriate Illumina sequencing adapters and a unique 12 bp Golay barcode in the reverse primer. PCR products were purified, normalized by DNA concentration and pooled into one library for sequencing on the Illumina MiSeq platform. Sequence data was deposited to NCBI under BioProject Accession number: PRJNA381082.

The sequencing data were processed and analyzed using the QIIME (v. 1.8) pipeline^70^. Overlapping 2 × 250 bp reads were stitched together using fastq-join allowing for 5% nucleotide difference in a minimum 200 bp overlap^71^. Reads that were unpaired, contained ambiguous bases or had a Phred quality score less than 20 were discarded. Sequences were clustered into operational taxonomic units (OTUs) at 97% sequence similarity using an open reference clustering scheme with uclust in QIIME^72^. OTUs that did not match a reference sequence in the Greengenes database (13_8 version available from http://greengenes.lbl.gov/) were retained and clustered *de novo* ^73^. Representative sequences were aligned using PyNAST and a taxonomic tree was constructed using FastTree^74, 75^. The resulting OTU table was filtered to remove OTUs containing less than five sequences, and the relative abundance of the bacterial taxa are reported as a percent of total sequences.

### Statistical analyses

Unpaired two-directional t test was used to analyze for statistical significance between the groups using GraphPad Prism version 6 (GraphPad Software, Inc. La Jolla, United States). The data are expressed as the mean ± standard deviation (SD). Differences were considered to be statistically significant when p < 0.05.

### Real-time quantitative PCR statistics

For intestinal tissue, data were analyzed using statistical software R (version 3.2.3; www.r-project.org). The statistical models were computed using R package nlme, and the contrasts with the corresponding p-values were obtained using R package multcomp. The data of each parameter (i.e. expression of each gene) were log-transformed and modeled using a linear model with terms for intestine part, treatment, and their interaction. The model used was a mixed model with a subject wise random intercept term or, if the mixed model could not be fit to the data, a generalized least squares model. The comparisons were performed using model contrasts, and the p-values were adjusted for multiple comparisons. The adjusted p values were computed from the joint normal or t distribution of the z statistics, which is the default method of the multcomp package^76^. Adjusted p-values < 0.05 were considered as statistically significant. For liver samples, the statistical analysis was done using two-directional unpaired t-test with GraphPad Prism version 6.0 as above.

Spearman correlation analysis was conducted for gene expression values and lipid parameters using GraphPad Prism version 6.

### Microbiome Sequence Analysis and Statistics

Alpha (within sample) and beta (between-sample dissimilarity) diversity were analyzed in QIIME with an OTU table rarefied at a depth of 24,279 sequences per sample. Alpha diversity was assessed by the Observed OTUs and Phylogenetic Diversity Whole Tree metrics and group comparisons were made using a non-parametric t-test with 1,000 Monte Carlo permutations^77^. Beta diversity was measured with unweighted and weighted UniFrac metrics^78^. The resulting distance matrix was visualized using a principal coordinate analysis (PCoA) plot with the top ten most abundant genera using the bi-plot function and EMPeror in QIIME^79^. The significance of sample clustering was assessed using permutational multivariate analysis of variation (PERMANOVA) with 1,000 permutations. Discriminate taxa between groups were determined using Mann-Whitney U test in QIIME. Spearman correlation analysis was conducted for microbial genera, lipid parameters and gene expression values that showed group differences using GraphPad Prism version 6. For all tests, p-values were subjected to the Benjamini-Hochberg false discovery rate (FDR) correction, and p ≤ 0.05 was considered a significant difference.

## Electronic supplementary material

Supplementary Dataset

Supplementary Dataset 1

## References

[1] Heidemann C (2008). Dietary Patterns and Risk of Mortality From Cardiovascular Disease, Cancer, and All Causes in a Prospective Cohort of Women. Circulation.

[2] Reiner Z (2011). The european society of cardiology and the european atherosclerosis society (ESC/EAS) guidelines on the management of dyslipidemia. Revista Espanola de Cardiologia.

[3] Alberti KGMM (2009). Harmonizing the metabolic syndrome: A joint interim statement of the international diabetes federation task force on epidemiology and prevention; National heart, lung, and blood institute; American heart association; World heart federation; International atherosclerosis society; And international association for the study of obesity. Circulation.

[4] Saydah SH, Fradkin J, Cowie CC (2004). Poor Control of Risk Factors for Vascular Disease among Adults with Previously Diagnosed Diabetes. Journal of the American Medical Association.

[5] Chapman MJ (2011). Triglyceride-rich lipoproteins and high-density lipoprotein cholesterol in patients at high risk of cardiovascular disease: Evidence and guidance for management. European Heart Journal.

[6] Papathanasopoulos A, Camilleri M (2010). Dietary Fiber Supplements: Effects in Obesity and Metabolic Syndrome and Relationship to Gastrointestinal Functions. Gastroenterology.

[7] Galisteo M, Duarte J, Zarzuelo A (2008). Effects of dietary fibers on disturbances clustered in the metabolic syndrome. The Journal of nutritional biochemistry.

[8] Cani PD, Delzenne NM (2009). The role of the gut microbiota in energy metabolism and metabolic disease. Current Pharmaceutical Design.

[9] Cordain L (2005). Origins and evolution of the Western diet: health implications for the 21st century. The American Journal of Clinical Nutrition.

[10] Milani C (2016). The human gut microbiota and its interactive connections to diet. Journal of Human Nutrition and Dietetics.

[11] Hooper LV, Littman DR, Macpherson AJ (2012). Interactions between the microbiota and the immune system. Science.

[12] Turnbaugh, P. J. *et al*. The effect of diet on the human gut microbiome: A metagenomic analysis in humanized gnotobiotic mice. *Science Translational Medicine* **1**, doi:10.1126/scitranslmed.3000322 (2009). 10.1126/scitranslmed.3000322 PMC2894525 20368178

[13] Hildebrandt MA (2009). High-Fat Diet Determines the Composition of the Murine Gut Microbiome Independently of Obesity. Gastroenterology.

[14] Canfora EE, Jocken JW, Blaak EE (2015). Short-chain fatty acids in control of body weight and insulin sensitivity. Nature Reviews Endocrinology.

[15] Murphy O (2001). Non-polyol low-digestible carbohydrates: food applications and functional benefits. British Journal of Nutrition.

[16] Beards E, Tuohy K, Gibson G (2010). A human volunteer study to assess the impact of confectionery sweeteners on the gut microbiota composition. British Journal of Nutrition.

[17] Herfel TM (2011). Polydextrose enrichment of infant formula demonstrates prebiotic characteristics by altering intestinal microbiota, organic acid concentrations, and cytokine expression in suckling piglets. Journal of Nutrition.

[18] Vester Boler BM (2011). Digestive physiological outcomes related to polydextrose and soluble maize fibre consumption by healthy adult men. British Journal of Nutrition.

[19] Schwab U, Louheranta A, Torronen A, Uusitupa M (2006). Impact of sugar beet pectin and polydextrose on fasting and postprandial glycemia and fasting concentrations of serum total and lipoprotein lipids in middle-aged subjects with abnormal glucose metabolism. European journal of clinical nutrition.

[20] Pronczuk A, Hayes KC (2006). Hypocholesterolemic effect of dietary polydextrose in gerbils and humans. Nutrition Research.

[21] Ravussin Y (2012). Responses of gut microbiota to diet composition and weight loss in lean and obese mice. Obesity.

[22] Cani, P. D. *et al*. Selective increases of bifidobacteria in gut microflora improve high-fat-diet-induced diabetes in mice through a mechanism associated with endotoxaemia. *Diabetologia* **50**, doi:10.1007/s00125-007-0791-0 (2007). 10.1007/s00125-007-0791-0 17823788

[23] Clavel T (2014). Intestinal microbiota in metabolic diseases: From bacterial community structure and functions to species of pathophysiological relevance. Gut Microbes.

[24] Ibarra, A., Astbury, N. M., Olli, K., Alhoniemi, E. & Tiihonen, K. Effect of polydextrose on subjective feelings of appetite during the satiation and satiety periods: A systematic review and meta-analysis. *Nutrients* **8**, doi:10.3390/nu8010045 (2016). 10.3390/nu8010045 PMC4728658 26784221

[25] Ibarra A, Astbury NM, Olli K, Alhoniemi E, Tiihonen K (2015). Effects of polydextrose on different levels of energy intake. A systematic review and meta-analysis. Appetite.

[26] Hooda S (2012). 454 Pyrosequencing reveals a shift in fecal microbiota of healthy adult men consuming polydextrose or soluble corn fiber. The Journal of Nutrition.

[27] Jakobsdottir, G., Xu, J., Molin, G., Ahrné, S. & Nyman, M. High-fat diet reduces the formation of butyrate, but increases succinate, inflammation, liver fat and cholesterol in rats, while dietary fibre counteracts these effects. *PLoS ONE* **8**, doi:10.1371/journal.pone.0080476 (2013). 10.1371/journal.pone.0080476 PMC3827442 24236183

[28] Lin, H. V. *et al*. Butyrate and propionate protect against diet-induced obesity and regulate gut hormones via free fatty acid receptor 3-independent mechanisms. *PLoS ONE* **7**, doi:10.1371/journal.pone.0035240 (2012). 10.1371/journal.pone.0035240 PMC3323649 22506074

[29] Tolhurst G (2012). Short-chain fatty acids stimulate glucagon-like peptide-1 secretion via the G-protein-coupled receptor FFAR2. Diabetes.

[30] Olli K (2015). Postprandial effects of polydextrose on satiety hormone responses and subjective feelings of appetite in obese participants. Nutrition Journal.

[31] Ibarra A (2017). Effects of polydextrose with breakfast or with a midmorning preload on food intake and other appetite-related parameters in healthy normal-weight and overweight females: An acute, randomized, double-blind, placebo-controlled, and crossover study. Appetite.

[32] Grootaert C (2011). Bacterial monocultures, propionate, butyrate and H2O2 modulate the expression, secretion and structure of the fasting-induced adipose factor in gut epithelial cell lines. Environmental Microbiology.

[33] Alex S (2013). Short-chain fatty acids stimulate angiopoietin-like 4 synthesis in human colon adenocarcinoma cells by activating peroxisome proliferator-activated receptor γ. Molecular and Cellular Biology.

[34] Aguilar EC (2014). Butyrate impairs atherogenesis by reducing plaque inflammation and vulnerability and decreasing NFκB activation. Nutrition, Metabolism and Cardiovascular Diseases.

[35] Zhang, X., Osaka, T. & Tsuneda, S. Bacterial metabolites directly modulate farnesoid X receptor activity. *Nutrition and Metabolism* **12**, doi:10.1186/s12986-015-0045-y (2015). 10.1186/s12986-015-0045-y PMC4657204 26604978

[36] Caz V (2015). Modulation of cholesterol-related gene expression by dietary fiber fractions from edible mushrooms. Journal of Agricultural and Food Chemistry.

[37] Sun Y (2015). Effect of short-chain fatty acids on triacylglycerol accumulation, lipid droplet formation and lipogenic gene expression in goat mammary epithelial cells. Animal Science Journal.

[38] Lu, Y. *et al*. Short chain fatty acids prevent high-fat-diet-induced obesity in mice by regulating g protein-coupled receptors and gut Microbiota. *Scientific Reports* **6**, doi:10.1038/srep37589 (2016). 10.1038/srep37589 PMC5124860 27892486

[39] Chambers ES (2014). Effects of targeted delivery of propionate to the human colon on appetite regulation, body weight maintenance and adiposity in overweight adults. Gut.

[40] den Besten G (2015). Short-Chain Fatty Acids Protect Against High-Fat Diet–Induced Obesity via a PPARγ-Dependent Switch From Lipogenesis to Fat Oxidation. Diabetes.

[41] Putaala H, Makivuokko H, Tiihonen K, Rautonen N (2011). Simulated colon fiber metabolome regulates genes involved in cell cycle, apoptosis, and energy metabolism in human colon cancer cells. Mol. Cell. Biochem..

[42] Fava F (2007). Effect of polydextrose on intestinal microbes and immune functions in pigs. British Journal of Nutrition.

[43] Conterno L, Fava F, Viola R, Tuohy KM (2011). Obesity and the gut microbiota: Does up-regulating colonic fermentation protect against obesity and metabolic disease?. Genes and Nutrition.

[44] Fleissner CK (2010). Absence of intestinal microbiota does not protect mice from diet-induced obesity. British Journal of Nutrition.

[45] Drover VA (2008). CD36 mediates both cellular uptake of very long chain fatty acids and their intestinal absorption in mice. Journal of Biological Chemistry.

[46] Tran TTT (2011). Luminal lipid regulates CD36 levels and downstream signaling to stimulate chylomicron synthesis. Journal of Biological Chemistry.

[47] Smith SJ (2000). Obesity resistance and multiple mechanisms of triglyceride synthesis in mice lacking Dgat. Nature Genetics.

[48] Lee B, Fast AM, Zhu J, Cheng JX, Buhman KK (2010). Intestine-specific expression of acyl CoA:diacylglycerol acyltransferase 1 reverses resistance to diet-induced hepatic steatosis and obesity in Dgat1 -/- mice. Journal of Lipid Research.

[49] Liu L (2011). DGAT1 deficiency decreases PPAR expression and does not lead to lipotoxicity in cardiac and skeletal muscle. Journal of Lipid Research.

[50] Zhang Y (2006). Activation of the nuclear FXR improves hyperglycemia and hyperlipidemia in diabetic mice. Proceedings of the National Academy of Sciences of the United States of America.

[51] Mencarelli A, Renga B, Distrutti E, Fiorucci S (2009). Antiatherosclerotic effect of farnesoid X receptor. American Journal of Physiology - Heart and Circulatory Physiology.

[52] Turnbaugh PJ (2009). A core gut microbiome in obese and lean twins. Nature.

[53] Turnbaugh PJ, Bäckhed F, Fulton L, Gordon JI (2008). Diet-Induced Obesity Is Linked to Marked but Reversible Alterations in the Mouse Distal Gut Microbiome. Cell Host and Microbe.

[54] Lim MY (2016). The effect of heritability and host genetics on the gut microbiota and metabolic syndrome. Gut.

[55] Duncan SH (2008). Human colonic microbiota associated with diet, obesity and weight loss. International Journal of Obesity.

[56] Schwiertz A (2010). Microbiota and SCFA in lean and overweight healthy subjects. Obesity.

[57] Hamilton MK, Boudry G, Lemay DG, Raybould HE (2015). Changes in intestinal barrier function and gut microbiota in high-fat diet-fed rats are dynamic and region dependent. American Journal of Physiology - Gastrointestinal and Liver Physiology.

[58] Martinez I (2009). Diet-induced metabolic improvements in a hamster model of hypercholesterolemia are strongly linked to alterations of the gut microbiota. Appl Environ Microbiol.

[59] Everard A (2014). Microbiome of prebiotic-treated mice reveals novel targets involved in host response during obesity. ISME Journal.

[60] Kalliomäki M, Collado MC, Salminen S, Isolauri E (2008). Early differences in fecal microbiota composition in children may predict overweight. American Journal of Clinical Nutrition.

[61] Stenman, L. K. *et al*. Probiotic With or Without Fiber Controls Body Fat Mass, Associated With Serum Zonulin, in Overweight and Obese Adults-Randomized Controlled Trial. *EBioMedicine* **13**, 190–200, doi:10.1016/j.ebiom.2016.10.036. 10.1016/j.ebiom.2016.10.036 PMC5264483 27810310

[62] Karlsson FH (2013). Gut metagenome in European women with normal, impaired and diabetic glucose control. Nature.

[63] Kim JH (2014). Hypolipidemic and antiinflammation activities of fermented soybean fibers from meju in C57BL/6 J mice. Phytotherapy Research.

[64] Shen W (2014). Intestinal and systemic inflammatory responses are positively associated with sulfidogenic bacteria abundance in high-fat-fed male C57BL/6J mice. Journal of Nutrition.

[65] Lecomte, V. *et al*. Changes in gut microbiota in rats fed a high fat diet correlate with obesity-associated metabolic parameters. *PLoS ONE* **10**, doi:10.1371/journal.pone.0126931 (2015). 10.1371/journal.pone.0126931 PMC4436290 25992554

[66] Baldwin J (2016). Table grape consumption reduces adiposity and markers of hepatic lipogenesis and alters gut microbiota in butter fat-fed mice. The Journal of nutritional biochemistry.

[67] Walker A (2014). Distinct signatures of host-microbial meta-metabolome and gut microbiome in two C57BL/6 strains under high-fat diet. ISME Journal.

[68] Lin, H., An, Y., Hao, F., Wang, Y. & Tang, H. Correlations of Fecal Metabonomic and Microbiomic Changes Induced by High-fat Diet in the Pre-Obesity State. *Scientific Reports* **6**, doi:10.1038/srep21618 (2016). 10.1038/srep21618 PMC4768318 26916743

[69] Caporaso JG (2010). QIIME allows analysis of high-throughput community sequencing data. Nature Methods.

[70] Caporaso JG (2010). QIIME allows analysis of high-throughput community sequencing data. Nature Methods.

[71] Aronesty, E. ea-utils: “Command-line tools for processing biological sequencing data” (2011).

[72] Edgar RC (2010). Search and clustering orders of magnitude faster than BLAST. Bioinformatics.

[73] DeSantis TZ (2006). Greengenes, a chimera-checked 16S rRNA gene database and workbench compatible with ARB. Applied and Environmental Microbiology.

[74] Price, M. N., Dehal, P. S. & Arkin, A. P. FastTree 2 - Approximately maximum-likelihood trees for large alignments. *PLoS ONE* **5**, doi:10.1371/journal.pone.0009490 (2010). 10.1371/journal.pone.0009490 PMC2835736 20224823

[75] Caporaso, J. G. *et al*. PyNAST: A flexible tool for aligning sequences to a template alignment. *Bioinformatics* **26**, 266–267, doi:10.1093/bioinformatics/btp636 (2010). 10.1093/bioinformatics/btp636 PMC2804299 19914921

[76] Hothorn T, Bretz F, Westfall P (2008). Simultaneous inference in general parametric models. Biom J.

[77] Faith DP (1992). Conservation evaluation and phylogenetic diversity. Biological Conservation.

[78] Lozupone C, Knight R (2005). UniFrac: A new phylogenetic method for comparing microbial communities. Applied and Environmental Microbiology.

[79] Vázquez-Baeza Y, Pirrung M, Gonzalez A, Knight R (2013). EMPeror: a tool for visualizing high-throughput microbial community data. GigaScience.

