# Supplementary material for: Polydextrose changes the gut microbiome and attenuates fasting triglyceride and cholesterol levels in Western diet fed mice
Source: Sci Rep. 2017 Jul 13;7:5294. doi: 10.1038/s41598-017-05259-3 (PMC5509720; doi:10.1038/s41598-017-05259-3)

**Polydextrose changes the gut microbiome and attenuates fasting triglyceride and cholesterol levels in western diet fed mice**

Ghulam Shere Raza^1*^, Heli Putaala^2*^, Ashley Hibberd^3^, Esa Alhoniemi^4^, Kirsti Tiihonen^2^, Kari Antero Mäkelä^1^, Karl-Heinz Herzig^1,5,6^

**Supplementary tables and figures**

**Supplementary table S1.** Gene expression primers; TaqMan gene expression assays used for a) intestinal and b) liver tissue analysis.

**Supplementary table S2.** Spearman correlation coefficients of liver gene expression as analyzed by real-time quantitative PCR with plasma triglycerides and cholesterol. Spearman correlation (r) values and only significant changes (p) are reported in the table.

**Supplementary Fig S1**. **a)** Liver, **b)** epididymal fat and **c)** caecal content weight. All the values are presented as mean+SD Significant (p<0.04) reduction in epididymal fat weight has been observed in WD+PDX compared to WD fed animals. Caecal content weight was significantly increased (p<0.001) WD+PDX fed animals compared to WD.

**Supplementary Fig S2.** Fecal fat content from a single pooled sample. The unstriped columns represent the fecal fat content at d1 and the striped columns represent the fecal fat content at experimental day 14. WD; western diet fed mice, WD+PDX; western diet fed mice which received PDX 75 mg/day twice daily po for 14 days.

**Supplementary Fig S3.** Gene expression analysis of liver a) *Hmgcr*, b) *Ldlr*, c) *Acot3*, d) *Prkaca*, e) *Prkaa*, f) *Acat1*, g) *Acot2*, h) *Cyp7a1*, i) *Slc27a2*, j) *Acot6*, k) *Cd36*, l) *Lpl*, m) *Ppargc1* and n) *Ppara* as analyzed by real-time quantitative PCR. There were only three genes showing tendency in significance as calculated with unpaired t-test between the groups: *Hmgcr*, *Ldlr* and *Acot3*. WD; western diet fed mice, WD+PDX; western diet fed mice which received PDX 75 mg/day twice daily po for 14 days.

Supplementary table S1

A)

| Gene Name | Symbol | Assay id |
| --- | --- | --- |
| Acyl-CoA synthetase long-chain family member 3 | *Acsl3* | Mm01255804_m1 |
| Acyl-CoA synthetase long-chain family member 5 | *Acsl5* | Mm01261083_m1 |
| CD36 antigen | *Cd36* | Mm01135198_m1 |
| Diacylglycerol O-acyltransferase 1 | *Dgat1* | Mm00515643_m1 |
| Fatty acid binding protein 2, intestinal | *Fabp2* | Mm00433188_m1 |
| Fibroblast growth factor 15 | *Fgf15* |  |
| Angiopoietin-like 4 | *Fiaf* | Mm00480431_m1 |
| Nuclear receptor subfamily 1, group H, member 4 | *Fxr* | Mm00436425_m1 |
| Niemann-Pick type C1 | *Npc1* | Mm00435300_m1 |
| NPC1-like 1 | *Npcl1* | Mm01191972_m1 |
| peroxisome proliferator activated receptor alpha | *Ppara* | Mm00440939_m1 |
| Peroxisome proliferative activated receptor, gamma, coactivator 1 alpha | *Ppargc1* | Mm01208835_m1 |
| Solute carrier family 10, member 2 | *Slc10a2* | Mm00488258_m1 |
| Ribosomal protein, large, P0 | *Rplp0* | Mm00725448_s1 |

B)

| Gene Name | Symbol | Assay id |
| --- | --- | --- |
| Acetyl-Coenzyme A acetyltransferase 1 | *Acat1* | Mm00507463_m1 |
| Acyl-CoA thioesterase 2 | *Acot2* | Mm01622461_s1 |
| Acyl-CoA thioesterase 3 | *Acot3* | Mm00652967_m1 |
| Acyl-CoA thioesterase 6 | *Acot6* | Mm01297748_m1 |
| CD36 antigen | *Cd36* | As in intestinal tissue assay |
| Cytochrome P450, family 7, subfamily a, polypeptide 1 | *Cyp7a1* | Mm00484150_m1 |
| 3-Hydroxy-3-methylglutaryl-Coenzyme A reductase | *Hmgcr* | Mm01282499_m1 |
| Lipoprotein lipase | *Lpl* | Mm01345523_m1 |
| Low density lipoprotein receptor | *Ldlr* | Mm01177349_m1 |
| Peroxisome proliferator activated receptor alpha | *Ppara* | As in intestinal tissue |
| Peroxisome proliferative activated receptor, gamma, coactivator 1 alpha | *Ppargc1* | As in intestinal tissue assay |
| Protein kinase, AMP-activated, alpha 1 catalytic subunit | *Prkaa1* | Mm01296700_m1 |
| Protein kinase, cAMP dependent, catalytic, alpha | *Prkaca* | Mm00660092_m1 |
| Solute carrier family 27 (fatty acid transporter), member 2 | *Slc27a2* | Mm00449517_m1 |
| Ribosomal protein, large, P0 | *Rplp0* | As in intestinal tissue assay |

Supplementary table S2

|  | *Acat* | *Acot3* | *Hmgcr* | *Ldlr* | *Lpl* | *Ppara* | *Ppargc1* | *Prkaa1* | *Slc27a2* |
| --- | --- | --- | --- | --- | --- | --- | --- | --- | --- |
| WD+PDX TG  Spearman r | -0,738 | -0,838 | -0,720 | -0,761 | -0,73 | -0,743 | -0,788 | -0,615 | -0,875 |
| WD+PDX TG  p | 0,012 | 0,002 | 0,015 | 0,008 | 0,013 | 0,011 | 0,005 | 0,046 | 0,001 |
| WD+PDX Chol  Spearman r | - | - | - | - | -0,7545 | - | - | - | - |
| WD+PDX Chol  p | - | - | - | - | 0,0098 | - | - | - | - |

Supplementary Fig S1.


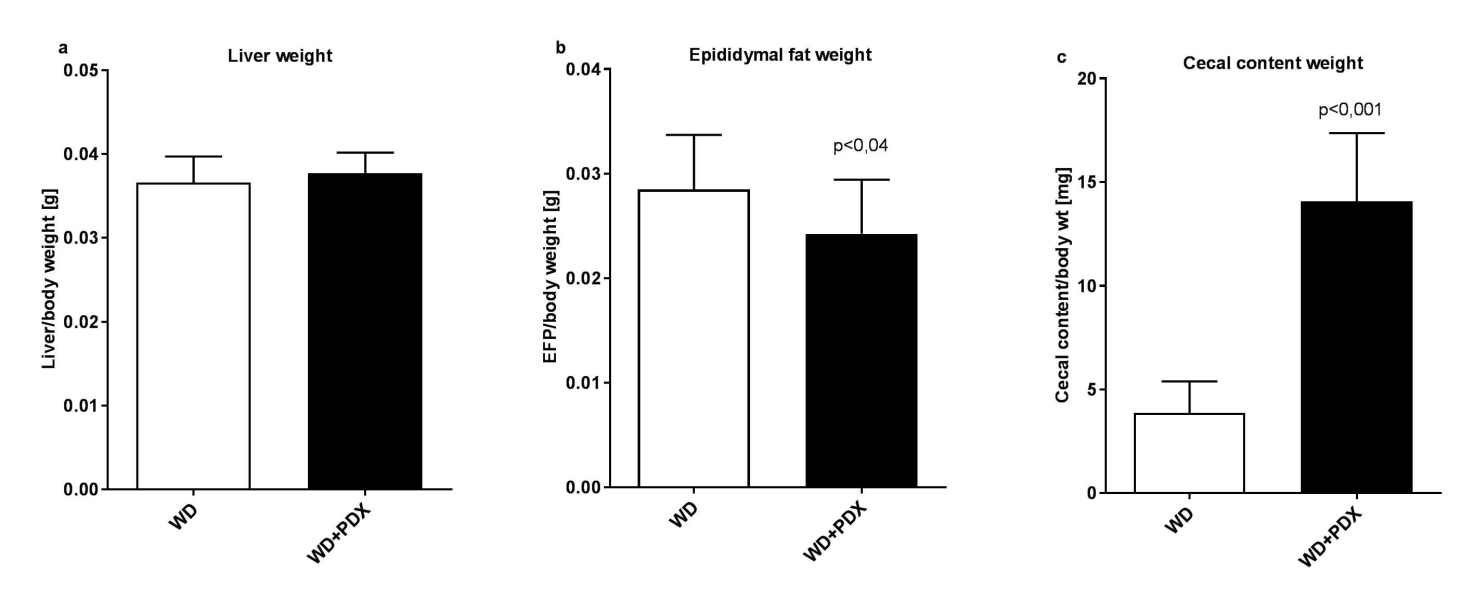


Supplementary Fig S2.


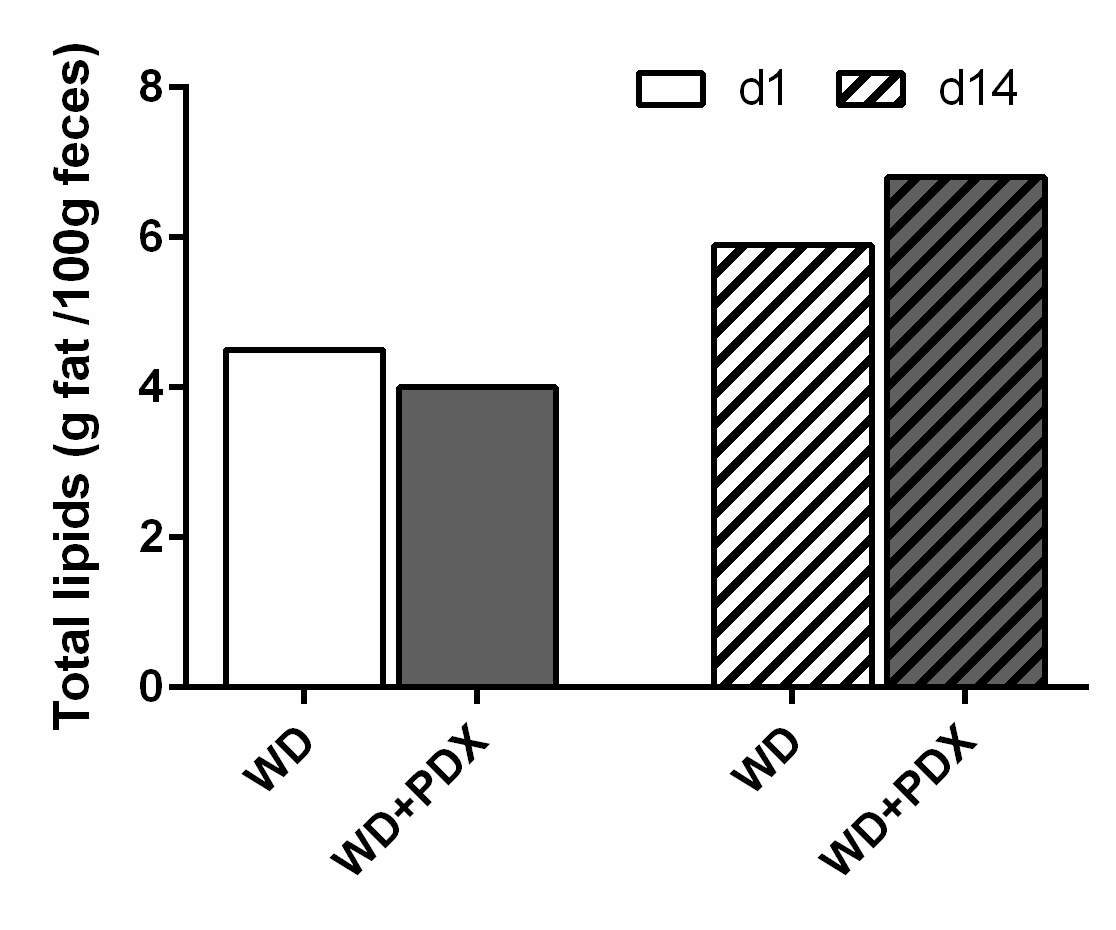


Supplementary Fig S3.


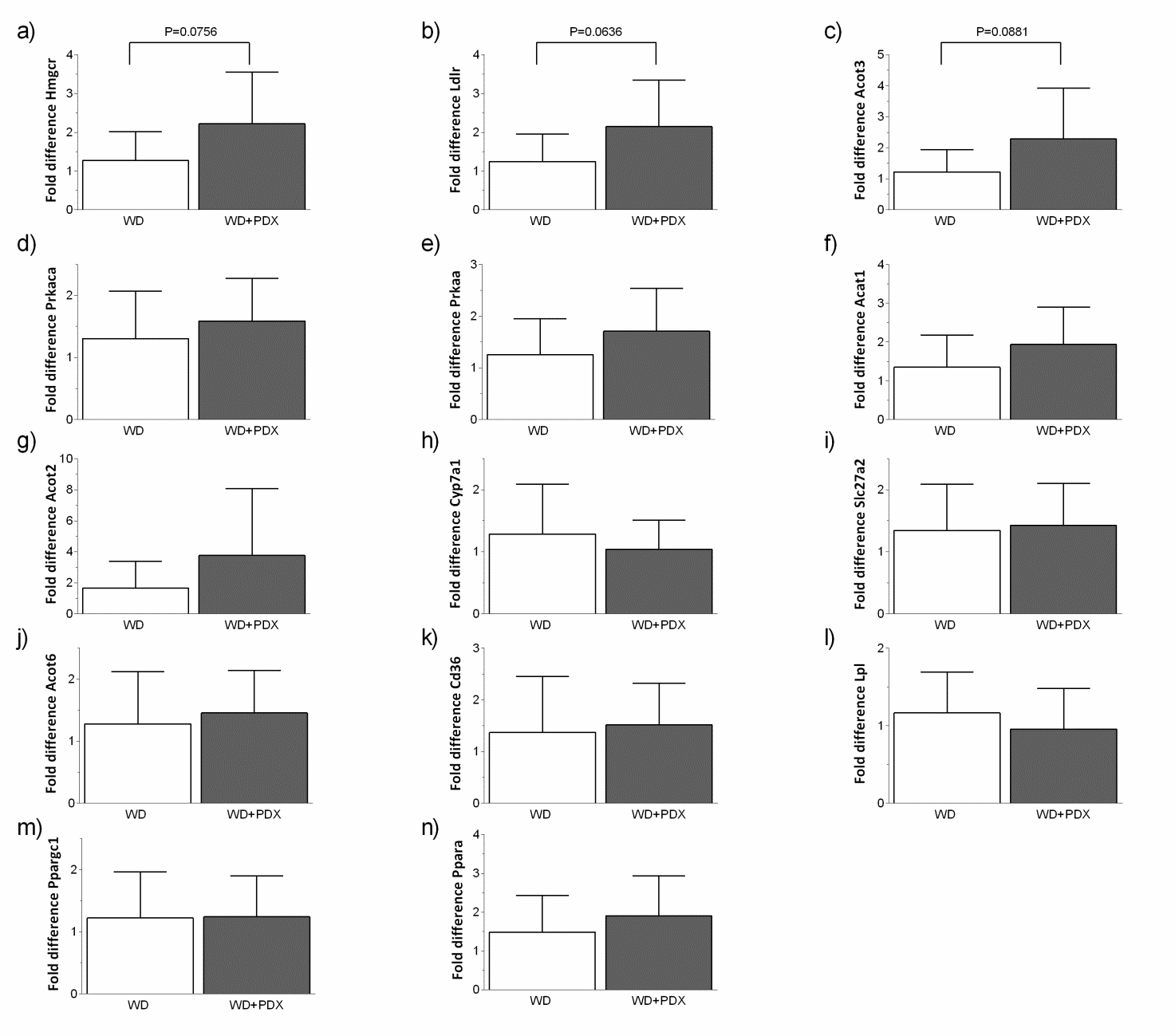

Supplement: Supplementary file 1 — Supplementary Dataset [file 41598_2017_5259_MOESM1_ESM.doc]
