# Supplementary material for: Polydextrose changes the gut microbiome and attenuates fasting triglyceride and cholesterol levels in Western diet fed mice
Source: Sci Rep. 2017 Jul 13;7:5294. doi: 10.1038/s41598-017-05259-3 (PMC5509720; doi:10.1038/s41598-017-05259-3)
Supplement: Supplementary file 2 — Supplementary Dataset 1 [file 41598_2017_5259_MOESM2_ESM.doc]

**Polydextrose changes the gut microbiome and attenuates fasting triglyceride and cholesterol levels in western diet fed mice**

Ghulam Shere Raza^1*^, Heli Putaala^2*^, Ashley Hibberd^3^, Esa Alhoniemi^4^, Kirsti Tiihonen^2^, Kari Antero Mäkelä^1^, Karl-Heinz Herzig^1,5,6^

**Supplemental Fig S4.** Alpha rarefaction curves generated for observed OTUs using 10 iterations for each step from the caecal microbiota of WD and WD+PDX mice. A depth of 24,279 sequences per sample was used for subsequent analyses. Group Means ± SD are shown.

**Supplemental Fig S5.** Principal coordinates analysis (PCoA) for unweighted UniFrac distance in caecal microbial communities from WD and WD+PDX mice. Sample clustering by diet is significant P< 0.001 (PERMANOVA).

Supplementary Fig S4.

Supplementary Fig S5.
